# Statin eligibility based on the ACC/AHA guidelines among Middle Eastern patients with diabetes mellitus presenting with acute myocardial infarction

**DOI:** 10.1016/j.amsu.2020.12.036

**Published:** 2020-12-29

**Authors:** Mohamad I. Jarrah, Muhannad J. Ababneh, Loai Issa Tawalbeh, Ayman J. Hammoudeh, Hanan M. Barukba, Ahmad Othman

**Affiliations:** a Department of Internal Medicine, Faculty of Medicine, Jordan University of Science and Technology, Irbid, Jordan; b Faculty of Nursing, Al al-Bayt University Al-Mafraq, P.O. Box: 130049, 25113, Jordan; c Department of Cardiology, and Coronary Computed Tomography Section, Istishari Hospital, Amman, Jordan

**Keywords:** ACC/AHA, Diabetes mellitus, Statin eligibility, Middle east

## Abstract

**Background:**

Statin eligibility based on the American College of Cardiology/American Heart Association cholesterol guidelines among patients with diabetes admitted with first time acute myocardial infarction has not been evaluated in the Middle East.

**Purpose:**

To assess statin eligibility for diabetic patients admitted with first time myocardial infarction in Jordan according to ACC/AHA guidelines.

**Methods:**

Consecutive patients admitted with a first acute myocardial infarction who were not taking statins, and had their serum lipoproteins measured upon hospital admission were enrolled in the study. Statin eligibility among patients with diabetes admitted with first time myocardial infarction was determined based on the ACC/AHA guidelines.

**Results:**

Of 774 patients enrolled, 292 (37.30%) had diabetes. Compared with non-diabetic patients, those with diabetes were females, older, more hypertension, more hypercholesterolemia, more triglycerides, more diastolic blood pressure, less smokers and less low density lipoprotein. Among patients with diabetes, 242 diabetic patients (82.9%) were statin eligible, including 20 (6.90%) for having high serum levels of low density lipoprotein cholesterol (LDL-C) >190 mg/dL, and 222 (76%) for being aged 40–75 years with LDL-C 70–189 mg/dL. No patient had a calculated atherosclerotic cardiovascular risk score ≥7.5%. On the other hand, 393 non-diabetic patients (81.3%) were statin eligible, including 41 (8.50%) for having high serum levels of low density lipoprotein cholesterol (LDL-C) >190 mg/dL, and 351 (72.80%) for being aged 40–75 years with LDL-C 70–189 mg/dL.

**Conclusions:**

Based on the ACC/AHA guidelines, the majority of patients with diabetes admitted with first acute myocardial infarction would have been eligible for statin treatment if they have LDL-c >190 mg/dl or aged 40–75 years old and they have their LDL 70–189 mg/gl. More efforts should be taken for patients who are female, older than 50 years, hypertensive, elevated diastolic blood pressure have hypercholesterolemia, and elevated triglycerides because of their significant association with diabetes.

## Highlights

• 83% of patients with diabetes mellitus presenting with MI, were statin eligible.
• 393 non-diabetic patients (81.3%) were statin eligible in the current sample.
• No diabetic or non-diabetic patients has a 10 year ASCVR score between 5 and 7.4
• Statin was eligible in the same proportion among diabetic and diabetic patients.

## 1 Introduction

Diabetes may lead to severe complications such as cardiac diseases, stroke, and death [1]. It also, may decrease life expectancy up to 15 years [2]. The high prevalence of diabetes and the difficulty of its health care are major reasons for high economic cost and burden [3]. Diabetes is a chronic disease that influences about 30.3 million people in the United States, or about 9.4% of the population with an estimated cost that exceeds 237 billion dollars for only direct medical care [4].

High levels of low-density lipoprotein cholesterol (LDL-C) and total serum cholesterol (TC) have been recognized as strong predictor for CVD mortality [5]. However, LDL-C and TC were decreased in the United States from 1988 to 2010 among persons both taking and not taking lipid‐lowering medications [6]. Statin therapy is the cornerstone for the management of high cholesterol and it was indicated to decrease the risk of CVD [7,8].

Using statin therapy according to current clinical practice guidelines is an important cardiovascular preventive measure that has been indicated by different clinical studies to decrease the cardiovascular mortality [9]. Statin is indicated or considered when the individual is at high or intermediate 10-year atherosclerotic cardiovascular risk (10 Y ASCVD).The level of this risk is determined based on the 2013 American College of Cardiology/American Heart Association (ACC/AHA) was used to determine the 10 Y ASCVD risk [10]. However, relying on absolute risk to decide statin eligibility may raise the number of patients who are eligible for statin.

Based on 2013 ACC/AHA guideline, diabetic patients over the age of 40 years were one the four major statin benefit groups that need intensive efforts to decrease CVD events [10]. Patients with diabetes aged between 40 and 75 years, LDL-C of 70–189 mg/dl (1.8–4.9 mmol/L) and without coronary artery disease (CAD) or stroke are perfect indications for statin as a primary prophylaxis. A moderate-intensity statin for diabetic patients over the age of 40 years as a primary prophylaxis was recommended [7]. Also, in secondary prevention, the use of statin is so important for patients with cardiac diseases or stroke [11]. High doses of statin are needed for the secondary prophylaxis of diabetic patients with CAD or at increased CVD risk such as those with abnormal LDL-C levels, smokers, hypertension, or albuminuria [12,13].

Based on American Heart Association guidelines (2018), patients aged 40–75 years with DM and an LDL-C level of ≥70 mg/dL (≥1.8 mmol/L), guidelines recommended to start moderate-intensity statin without calculating 10-year ASCVD risk. In addition, adults aged 40–75 years without DM and with LDL-C levels ≥70 mg/dL (≥1.8 mmol/L) at a 10-year ASCVD risk of ≥7.5%, the guidelines recommended to start a moderate-intensity statin. In adults aged 40 to 75 without DM and 10-year risk of 5%–19.9%, this support the initiation of statin therapy. Finally, adults aged 40–75 years without DM and with LDL-C levels ≥70–189 mg/dL (≥1.8–4.9 mmol/L), at a 10-year ASCVD risk of ≥7.5%–19.9%, and if a decision about statin therapy is in doubt, consider determining coronary artery calcium [13].

Based on 2013 ACC/AHA guideline, new changes were indicated to the model of CVD risk assessment in aspects like family history exclusion, stroke risk inclusion, and lowering high-risk threshold to ≥7.5% instead of ≥10% [14]. Several studies have been conducted to evaluate accuracy and efficiency of recommendations provided in the ACC/AHA guidelines [15].

Different studies have revealed inconsistent results regarding the proportion of individuals who would or would not be eligible for statin therapy using the current treatment guidelines. Result from Middle East indicated that 88% [16] and 39% [17] of participants were statin eligible according to the 2013 ACC/AHA guideline. Another study was conducted in which the cohort ranged from 1,993,755 members in 2009 to 2,440,429 in 2015. Using statin were constant for adults with ASCVD (2009 78%; 2015 80%), LDL-C ≥ 190 mg/dL (2009 45%; 2015 44%), and diabetic patients (2009 74%; 2015 73%), but elevated for patients with 10-year ASCVD risk ≥ 7.5% (2009 36%; 2015 47%) [18]. However, another study indicated that the 7.7 million with diabetes out of 12.4 million are eligible for statin treatment [19]. The application of the 2013 ACC/AHA cholesterol treatment guideline is likely to differ by statin benefit group. Statin eligibility for diabetic compared with non-diabetic individuals based on 2013 American College of Cardiology/American Heart Association cholesterol guidelines is not well studied. Therefore, this study aimed to assess statin eligibility among patients with diabetes mellitus admitted with first time myocardial infarction based on the 2013 ACC/AHA guidelines.

## 2 Material and methods

### 2.1 Study design

A descriptive-correlational, retrospective design was used to achieve the purpose of the current study.

### 2.2 Sample and sampling technique

This study analyzed statin eligibility among patients with diabetes who were admitted with first time AMI. These patients had no previous history of ASCVD, no present or past use of statin therapy, and their serum lipoprotein levels were measured at hospital admission. Consecutive patients admitted with a first-time AMI to tertiary care centers in Jordan from April 2018 to June 2019 were assessed for potential inclusion in the study. These health care centers were distributed in north, middle and south of Jordan. These centers were classified as academic, public and private hospitals.

Sample size was calculated using G* power software. Using 0.90 power level, an alpha 0.05 and an effect size of 0.25 for independent sample *t*-test, a minimum of 676 patients need to participate in the current study. To overcome the problem of incomplete data, an additional 134 participants were added to have 800 participants.

Acute myocardial infarction was classified into: (a) ST-segment elevation MI (STEMI), and diagnosed by the occurrence of heart ischemic chest pain, ST-segment elevation of ≥2 mm in at least 2 leads in ECG, and positive cardiac enzymes, (b) non-ST-segment elevation MI, that determined by the presence of ischemic chest pain, ST-segment depression, inverted T wave, or normal ECG and positive cardiac enzymes.

Participants were considered diabetic when they were: formerly diagnosed by a physician, using anti-diabetic drugs, or had fasting plasma sugar (FBS)≥126 mg/dL, haemoglobin A1C level ≥6.5%, or random plasma sugar (RBS) ≥200 mg/dL with typical symptoms of hyperglycemia. Lipid profile was measured: serum levels of total, high-density lipoprotein (HDL) and LDL-cholesterol were measured also at hospital admission.

Arterial hypertension was determined based on the following criteria: diagnosis made by a physician, use of anti-hypertension drugs, or frequent systolic blood pressure (SBP) and/or diastolic BP (DBP) measurements ≥140/90 mm Hg throughout the hospital stay. Present cigarette smoking was identified as smoking one or more cigarettes per day for the last year. Family history of premature CAD were determined as CAD in male first level relative aged <55 years, or in female first level relative aged <65 years; and age (men ≥45 years; women ≥55 years).

Statin eligibility among the diabetic and non-diabetic individuals was determined based on the 2013 ACC/[10]. Participants were considered statin eligible according to the 2013 ACC/AHA guidelines if LDL-C levels ≥190 mg/dL, diabetes with age 40–75 years and LDL-C levels 70–189 mg/dL, or 10-year ASCVD risk score ≥7.5%. Statin is considered for individuals with risk score between 5% and 7.4%, and it was not recommended for individuals with risk score <5% [10]. Same guidelines were used for non-diabetic individuals.

The 10-year risk of ASCVD was determined using a web-based application presented at http://www.cvriskcalculator.com/using clinical and laboratory variables that contain age (40–79 years), SB.P (200-90 mm Hg), DB.P (30–140 mm Hg), serum TC (130–320 mg/dL) and HDL-C (20–100 mg/dL) [21]. To estimate the risk for individuals with value(s) beyond these ranges, the values were modified to be equivalent to the minimum or maximum value of that variable, i.e., cholesterol value of 340 mg/dL was approximated to 320 mg/dL. Informed consent was obtained from each participant and the Institutional Review Board of each participating hospital approved the study.

### 2.3 Data Collection procedure

First the ethical approval to conduct the study was obtained from the Institutional Review Board (IRB) of the King Abdullah University Hospital (KAUH). Research registry was done (UIN: Researchregistry6297) and the hyperlink is https://www.researchregistry.com/browse-the-registry#home/registrationdetails/5fbf95b3245846001ca4bb54/. This study was conducted in compliance with the ethical standards per Helsinki declaration. The research registry number is 6297. Trained data collectors (medical residents) started to collect the relevant data from the eligible patients. The patients, who were eligible (first time AMI, with no present or past use of statin), agreed to participate and provided the written informed consent, were provided with full information about the study and its purpose. A cover letter containing a description of the study, the participant's ethical and legal rights, and the researcher's contact information were provided for each participant. The data regarding lipid profile, blood pressure, body mass index, and ECG were collected upon hospital admission. In addition, other demographic and risk factors like age and gender, DM, hypertension, hypercholesterolemia, smoking, and family history of cardiovascular diseases, were collected during the interview at admission. The diagnosis of AMI was confirmed by the cardiologist in each participating hospital. No complications or any adverse events were detected during the study. Small blood sample were taken for lipid and sugar analysis. Each patient was monitored carefully during the procedure by medical resident.

### 2.4 Statistical analysis

Descriptive statistics mean (M), standard deviation (SD) or median for continuous variables, frequency (percentage) for categorical variables were used to describe the sample characteristics and the clinical variables measured in the study. Pearson's chi‐square was used to assess the significant association between selected demographic, clinical variables and diabetes. In addition, independent *t*-test and Mann-Whitney test were used to assess the significant difference between diabetic and non-diabetic in terms of selected clinical variables. A p-value of less than 0.05 was considered statistically significant. This work has been reported in line with the STROCSS criteria [21].

## 3 Results

### 3.1 Sample characteristics

Eight-hundred patients with first time AMI were recruited. However, 26 patients have incomplete data to have seven-hundred and seventy-four were in the final sample size. The mean age for them was 55 years (SD = 11.34). Results showed that almost 85% of the sample (n = 654) were males, 59% has hypertension (n = 458), three-quarters of the sample has hypercholesterolemia (n = 585), more than one-third is diabetic (n = 292), and almost 39% of the sample is smoker (n = 300). In addition, results revealed that nearly, 59% of the sample has a family history of CVDs, more than two-third has a history of STE-MI (n = 548). The majority of the patients (98%) were undergone cardiac CATH (n = 752), PCI was performed for 93% of the sample (n = 719) and only 1% of patients were undergone CABG (n = 8).

Results showed that the mean TC was 187.55 (SD = 52.11), and the mean for the LDL was 125 (SD = 46.00). Results indicated that the mean HDL was 37.39 (SD = 10.81), while the mean TGs was 188.40 (157.16). The mean systolic B.P was 129.06 mmgh (SD = 21.00), while the mean diastolic B.P was 77.58 mmgh (SD = 11.89). The mean body mass index (BMI) was 28.38 (SD = 4.69). Results of descriptive analysis are presented in Table 1.

**Table 1:** Sample Characteristics: demographics and the clinical factors for participants (N = 774).

Variables	Actual Range	M (SD)	N	%
Age (Years)	24–94	055.04 (11.34)	774	100
Total Cholesterol (TC)	65–464	187.55 (52.11)	774	100
Triglycerides (TGs)	29–2136	188.40 (157.20)	774	100
High Density Lipoprotein (HDL)	7–98	037.39 (10.81)	774	100
Low Density Lipoprotein (LDL)	11–376	125.76 (46.00)	774	100
Body Mass Index (BMI)	15.70–53.00	028.37 (04.69)	774	100
Systolic Blood Pressure	70–225	129.06 (21.00)	774	100
Diastolic Blood Pressure	40–120	077.58 (11.89)	774	100
Age				
<50 years			278	35.90
≥50 years			496	64.10
Gender				
Male			654	84.50
Female			120	15.50
Diabetes Mellitus (DM)				
Yes			292	37.30
No			482	62.30
Hypertension (HTN)				
Yes			316	40.80
No			458	59.20
Hypercholestrolemia				
Yes			189	24.40
No			585	75.60
Smoking				
Yes			474	61.20
No			300	38.80
MI-Type				
STEMI			548	70.80
Non-STEMI			226	29.20
Family history of cardiovascular diseases				
Yes			320	41.30
No			454	58.70
CATH				
Yes			752	97.20
No			22	2.80
Percutaneous Coronary Intervention (PCI)				
Yes			719	92.90
No			55	7.10
Coronary Artery Bypass Graft (CABG)				
Yes			8	1.00
No			766	99.00

### 3.2 Statin eligibility

Analysis showed that 242 diabetic patients (82.9%) were statin eligible including: (1) 20 diabetic patients (6.90%) with LDL-C≥190 mg/dL, (2) 222 diabetic patients (76.00%) whose age ranged 40–75 years and LDL-C levels 70–189 mg/dL, and (3) no diabetic patients (0%) with 10-year risk score of ≥7.5%. However, analysis showed that 393 non-diabetic individuals (81.3%) were statins eligible including: (1) 41 non-diabetic individuals (8.50%) with LDL-C≥190 mg/dL, (2) 351 non-diabetic individuals (72.80%) whose age ranged 40–75 years and LDL-C levels 70–189 mg/dL, and (3) no non-diabetic individuals (0%) with 10-year risk score ≥7.5%.

**Fig. 1:**
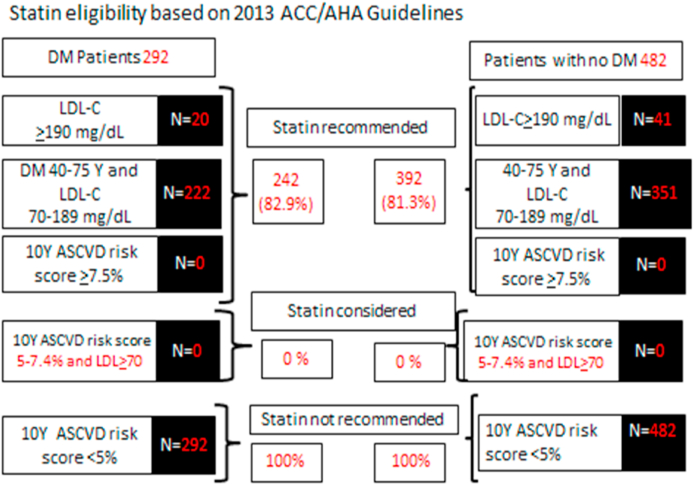
Statin eligibility based on 2013 ACC/AHA Guidelines.

**Fig. 2:**
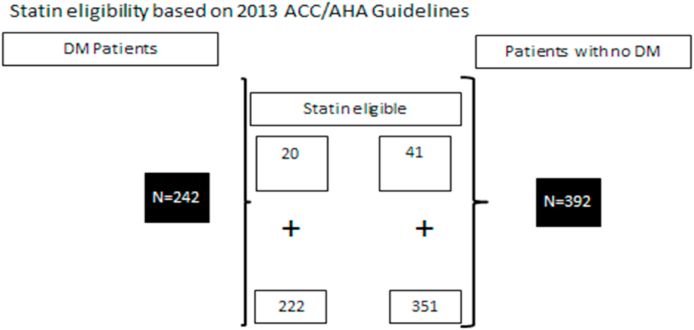
Statin eligibility based on 2013 ACC/AHA Guidelines.

Furthermore, statin would have been not considered for any diabetic and non-diabetic patients since no one has 10-year risk score of 5–7.5% based on this criterion (10-year risk score of 5–7.5%). In addition, statins would have been not indicated in all patients either diabetic or non-diabetic since all has 10-year risk score <5% based on this criterion (10-year risk score <5%). Statin eligibility was determined for 774 individuals; 292 diabetic patients and 482 non-diabetics (Fig. 1 and Fig. 2).

### 3.3 The association and the differences between selected demographic factors, clinical factors and D.M

To assess the association between selected demographic factors, clinical factors and D.M, chi-square test was applied as showed in Table 2. Results showed that there was a significant association between age X^2^ (1) = 9.44, P = 0.002, gender X^2^ (1) = 21.68, P < 0.001, HTN X^2^ (1) = 63.42, P < 0.001, smoking X^2^ (1) = 19.24, P < 0.00, hypercholesterolemia X^2^ (1) = 22.85, P < 0.001, PCI X^2^ (1) = 5.97, P = .01 and DM. Results indicated that patients aged ≥ 50 years are more likely to be diabetic (70.90%) than to be non-diabetic (60%). This indicated that compared with non-diabetic patients, those with diabetes mellitus were females (22%), older (70.9%), more hypertension (58.90%), more hypercholesterolemia (33.90%) and less smoker (51.40%) and more PCI (9590%) as indicated in Table 2. However, no significant association was found between MI type P = 0.35, family history of CVD P = 0.06, performing cardiac CATH P = 0.65, performing CABG P = 0.48, using of Ezitrol P = 0.16, using beta blockers P = 0.94, Aspirin P = 0.10, Aldactone P = 0.45 and DM. Table 2 showed that 774 are admitted with first time AMI, 548 (70.80%) out of them are presented with STEMI and 226 (29.20%) are presented with non-STEMI. Also, results showed that 292 (37.7%) patients with MI have DM, while 482 (62.30%) patients with MI did not have DM.

**Table 2:** Chi-square to examine the association between selected demographics, clinical factors and diabetes mellitus (N = 774).

	Diabetes Mellitus		P value
Demographic and clinical variable	No (n = 482)	Yes (n = 292)	
Age			0.002
<50 years (n = 278)	193 (40.00%)	85 (29.10)	
≥50 years (n = 496)	289 (60.00%)	207 (70.90)	
Gender			<0.001
Male (n = 654)	430 (89.20)	224 (76.70)	
Female (n = 120)	052 (10.80)	068 (22.30)	
MI-Type			0.34
STEMI (n = 548)	347 (71.90)	201(68.80)	
Non-STEMI (n = 226)	135 (28.10)	091 (31.20)	
Hypertension (HTN)			<0.001
No (n = 458)	338 (70.10)	120 (41.10)	
Yes (n = 316)	144 (29.90)	172 (58.90)	
Hypercholesterolemia			<0.001
No (n = 585)	392 (81.30)	193 (66.10)	
Yes (n = 189)	90 (18.70)	99 (33.90)	
Smoking			<0.001
No (n = 300)	158 (32.80)	142 (48.60)	
Yes (n = 474)	324 (67.20)	150 (51.40)	
Family history of CVDs			0.06
No (n = 454)	295 (61.20)	159 (54.5%)	
Yes (n = 320)	187 (38.80)	133 (45.50)	
CATH			0.56
No (n = 22)	15 (03.10)	7 (02.40)	
Yes (n = 752)	467 (96.90)	285 (97.60)	
PCI			0.01
No (n = 54)	42 (08.70)	12 (04.10)	
Yes (n = 719)	439 (91.30)	280 (95.90)	
CABG			0.48 (Fishers)
No (n = 766)	478 (99.20)	288 (98.60)	
Yes (n = 8)	4 (00.80)	4 (01.40)	
Ezitrol			0.16 (Fishers)
No (n = 769)	477 (99.00)	292 (100.0)	
Yes (n = 5)	5 (01.00)	0 (00.00)	
Beta-blockers			0.94
No (n = 263)	164 (34.20)	99 (33.90)	
Yes (n = 509)	316 (65.80)	193 (66.10)	
Aspirin			0.10
No	2 (00.40)	2 (00.70)	
Yes	480 (99.60)	290 (99.30)	
Aldactone			0.45
No	479 (99.40)	289 (99.00)	
Yes	3 (00.60)	3 (01.00)	

CVDs: cardiovascular diseases, PCI: Percutaneous coronary intervention, CABG: Coronary Artery Bypass Graft, Fisher exact test is reported.

Independent *t*-test showed that there was a statistically significant difference in Dia.B.P mean score t(772) = - 2.23, P < 0.02, between diabetic patients (M = 78.80, SD = 11.66) and non-diabetic patients (M = 76.84, SD = 11.97). This indicated that diabetic patients scored significantly in D.B.P higher than non-diabetic patients. Results of *t*-test are indicated in Table 3.

**Table 3:** Independent *t*-test to assess difference in selected clinical variables between diabetic and non-diabetic patients (N = 774).

Variable	M(SD)	t	df	P	CI
Diastolic blood pressure		−2.23	772	0.02	−3.69–−0.23
Diabetic patients	78.80 (11.66)				
Non-diabetic patients	76.84 (11.97)				

Mann-Whitney test showed that no statistically significant difference in the mean ranks of sys.B.P, HDL and TC between diabetic and non-diabetic patients. However, results revealed that the mean ranks for LDL for diabetic patients are statistically significant 368.18 (U = 61950, N1 = 292, N2 = 482, two-tailed, P = 0.005) and lower than the mean ranks for non-diabetic patients 404.97. In addition, results indicated that mean ranks for TGs for diabetic patients are statistically significant 409.52 (U = 69423, N1 = 292, N2 = 482, two-tailed, P = 0.03) and higher than the mean ranks for non-diabetic patients 374.16. This indicated that compared with non-diabetic patients, those with DM have more triglycerides, more diastolic blood pressure and less low density lipoprotein. Results of Mann-Whitney *t*-test are indicated in Table 4.

**Table 4:** Mann-Whitney *t*-test to assess the difference in selected clinical variables between diabetic and non-diabetic patients (N = 774).

Variable	Mean Rank	Mean (SD)	P value
Total Cholesterol (TC)			
Diabetic patients	368.18	185.41 (56.84)	0.06
Non-diabetic patients	399.20	188.84 (49.03)	
Triglycerides (TGs)			
Diabetic patients	409.52	206.49 (206.15)	0.03
Non-diabetic patients	374.16	187.11 (117.05)	
High Density Lipoprotein (HDL)			
Diabetic patients	380.61	37.14 (11.14)	0.50
Non-diabetic patients	391.67	37.54 (10.62)	
Low Density Lipoprotein (LDL)			
Diabetic patients	358.66	121.80 (48.94)	0.005
Non-diabetic patients	404.97	128.16 (44.01)	
Systolic blood pressure			
Diabetic patients	402.41	130.31 (20.54)	0.15
Non-diabetic patients	378.47	128.30 (21.26)	

## 4 Discussion

The major finding of the current study indicated that most of diabetic patients (83%) and non-diabetic individuals (81%) are statin eligible based on the 2013 ACC/AHA guidelines. Based on these guidelines, statin eligibility among our participants was higher than that at different international studies [18,19,22]. One study in USA was conducted to assess trends in statin use before and after application of the ACC/AHA guideline. A retrospective design was conducted with annual cohorts from 2009 to 2015 for the individuals aged ≥21 years. Results indicated that statin use for adults patients with diabetes and ranged from (2009 74%; 2015 73% [18]. In addition, same guidelines were assessed among 12.4 million adult's patients with diabetes. Results showed that almost 63% of the diabetic patients were statin eligible [19].

Moreover, data were used from the National Health and Nutrition Examination Surveys of 2005–2010, to assess statin eligibility based on the 2013 ACC/AHA guidelines in a population-based sample from the U.S. Results showed that statin was eligible among 48.6% of the adult's participants without CVD [23]. More recent National Health and Nutrition Examination Surveys of 2007–2012 were used to assess statin use among adults aged 21–79 years based on the 2013 ACC/AHA guidelines. Results showed that 25.5% of the participants were statin eligible [24].

The high proportion of statin eligibility among our sample in the current study could be explained by many factors. First, the high prevalence of diabetes among the sample in the current study which is consistent with different international studies [[25], [26], [27]]. In addition, 28% of the whole sample was diabetic, aged 40–75 years and LDL-C was between 70 and 189 mg/dl compared with 6% [18]. Moreover, this subgroup consisted 76% (n = 222) of the diabetic patients and 73% (n = 351) of the non-diabetic individuals whom were eligible for statin therapy.

Second, the high values of B.P, blood sugar, LDL-C, total cholesterol and TGs and low levels of HDL-C among our sample and in Middle East [17] compared with other international studies [28] may explain the need for more statin use among diabetic and non-diabetic in the current study. However, results indicated that no patients either diabetic or non-diabetic were considered statin eligible based on the 10 Y ASCVD risk score of ≥7.5%. This finding contradicted most of the previous studies in which the great majority of patients, were statin eligible, based on 10 Y ASCVD risk score ≥7.5% [18,23,24,30].

The results also showed that statin was not considered for either diabetic or non-diabetic based on the 10 Y ASCVD risk score between 5 and 7.4% and LDL≥70. This result is inconsistent with other findings that indicated a high proportion of individuals who are statin eligible based on 10 Y ASCVD risk score between 5 and 7.4% [[31], [32], [33], [34]]. The results also showed that statin was not recommended for either diabetic or non-diabetic based on the 10 Y ASCVD risk score between <5%. This result confirms the 2013 ACC/AHA guidelines that indicated an individual with a 10‐year CVD risk <5% is not recommended to receive pharmacological treatment to lower the serum cholesterol level [10].

Results of the current study showed that statin also was eligible in the majority of non-diabetic individuals based on ACC/AHA guidelines. This contradicts previous findings in which the ratio of statin eligibility was lower among non-diabetic compared with diabetic in different international studies [23,24] compared with the current study. This indicated that statin was eligible in the same proportion among diabetic as non-diabetic individuals. This could be explained that non-diabetic individuals in the current study were more likely to be smoker and have a significantly higher level of LDL-C than diabetic patients. Also, LDL-C elevation is a significant indication for statin therapy as reported in different studies [[10], [11], [12], [13]]. Although, we have to consider that relying on absolute risk may increase the number of patients who are eligible for statin therapy. Accordingly, further studies are recommended to address the statin eligibility among diabetic versus non-diabetic patients using a larger sample from different areas in Jordan. These studies may help clarify the factors which affect statin eligibility among diabetic and non-diabetic individuals.

Using A cross-sectional descriptive design may limit the external validity of the result. It is very important to assess statin eligibility among diabetic versus non-diabetic individuals in Middle East using experimental longitudinal study on a larger heterogeneous sample to enhance the generalizability of the findings. In addition, it would be beneficial to replicate the study comparing the impact of different guidelines on statin eligibility among diabetic versus non-diabetic. Moreover, the applications of the 2013 ACC/AHA guidelines in Middle East population who have DM may be affected by different variables age, gender, and cholesterol level. It is recommended to replicate the study controlling for these factors that would provide a specific standards to help determine Statin eligibility among diabetes.

Selection bias is a potential bias in the current study. However, different actions were implemented to reduce the potential bias which include; data were gathered trained data collectors, standardized information were given to all patients who participated in the current study, and all potential patients were examined for their eligibility. In addition all assumptions of statistical procedures were assured and met like normality of distribution and levene test value for independent *t*-test.

## 5 Conclusion

The impact of the 2013 American College of Cardiology/American Heart Association guidelines was clear and results showed that statin was eligible in the great majority of participants with almost the same percentage among diabetic and diabetic individuals. However, statin was not recommended for either diabetic or non-diabetic who 10 year ASCVR score below <7.4. Female patients, older than 50 years, hypertensive, and elevated diastolic blood pressure have hypercholesterolemia, and elevated triglycerides are of a major concern because of their significant association with diabetes.

## Provenance and peer review

Not commissioned, externally peer-reviewed.

## Funding

This study received no Funds.

## Author contribution

Study design: Mohamad I Jarrah, Muhannad J Ababneh, Loai Tawalbeh, Ayman J. Hammoudeh, Hanan M barukba, Ahmad R Abdel Rahman. Data Collection: Mohamad I Jarrah, Hanan M barukba, Ahmad R Abdel Rahman. Data analysis: Mohamad I Jarrah and Loai Tawalbeh. Writing the manuscript: Mohamad I Jarrah, Muhannad J Ababneh, Loai Tawalbeh, Ayman J. Hammoudeh, Hanan M barukba, Ahmad R Abdel Rahman.

## Ethical approval

The ethical approval to conduct the study was obtained from the Institutional Review Board (IRB) of the King Abdullah University Hospital (KAUH).

## Declaration of competing interest

No conflicts of interests.
